# Can tibial tantalum cones eventually eliminate the adjuvant use of metallic augments for AORI type 2B/3 metaphyseal defects??—A novel surgical technique and case series

**DOI:** 10.1016/j.ijscr.2018.09.028

**Published:** 2018-09-23

**Authors:** Mohit M. Kukreja, Todd V. Swanson

**Affiliations:** a Swanson Hip and Knee Center of Excellence and Research Institute Desert Orthopaedic Center, Las Vegas, NV, USA; b Desert Orthopedic Center & Swanson Hip/Knee Research Foundation, 2800 E.Desert Inn, Suit 100, Las Vegas, NV, 89121, USA

**Keywords:** Tantalum cones, Metaphyseal bone loss, Metallic augments, Tibia

## Abstract

**Background:**

For extensive metaphyseal defects, the use of tantalum cones is usually combined with adjuvant stems which may be cemented/cementless and metallic bone augments for additional stability. A Tibial baseplate-Cone construct with proud tibial cones and without metallic augments has been described for AORI type 2B/3 tibial defects.

**Methods:**

A case series analysis of 6 patients with AORI type 3 defects treated with unstepped proud Tantalum tibial metaphyseal cones without metallic wedges/full width augments. A follow-up analysis done with clinico-radiographic interpretation.

**Results:**

100% osteointegration noted in final radiographs of all patients at an average follow-up of 4.1 years. Preoperative average ROM/KSS of 75/49 improved to postoperative ROM/KSS of 104/79. Outcomes were interpreted as excellent in 50% of cases and good in the remaining 50%.

**Conclusion:**

The “Tibial base plate-cone without augments (BCCA)”type of a construct may offer a valid long term advantage over the Tibial base plate-Augment-Cone combination in massive tibial bone defects. Simply building up the tantalum cone to the native joint line position by increasing cone height can exclude use of augments or wedges.

## Highlights

• A Tibial baseplate-Cone construct with proud tibial cones and without metallic augments has been described for AORI type 2B/3 tibial defects.
• The technique mainly involves that the host bone be prepared for the reception of the Cone with a high-speed burr. The Cone that would press-fit to obtain maximum axial stability but at the same time reconstitute the joint line to the closest native position without use of full-width augments/wedges.
• Morsellized allograft bone/demineralized bone matrix (DBX; Synthes Westchester Pennsylvania) used to fill in any minute voids between the host bone and the cone so as to make efforts for maximal host bone-cone contact area.
• Cementing all the tibial stems upto the diaphysis is advised.
• A stronger Tantalum cone-cement interdigitation around the stem due to a longer contact area between them because of exclusion of metallic augments seems a valid advantage.
• Also, the tibial tray baseplate sitting directly on the cone with interfingering cement could be another parallel boost to the construct stability over the base plate-augment interface with smooth metallic surfaces on both sides.
• The “Tibial base plate-cone without augments (BCCA)”type (Swanson’s technique) of a construct may offer a valid long term advantage over the Tibial base plate-Augment-Cone combination in massive tibial bone defects. Larger studies are expected to validate the proposed technique and its long-term advantage.

## 1 Introduction

Massive bone defects presenting concomitantly with component loosening, subsidence and osteolysis are a routine intraoperative finding during Revision TKA. Achieving stability of the construct in the presence of such bone deficiencies is a matter of debate and holds prime importance.

Management of bone loss in a Revision TKA represents a humongous challenge. The bone defect may result from the design of the primary prosthesis used, the actual initial disease process, the technical errors done during the index operation or iatrogenically while removing the prosthesis [1].Axial and rotational stability of implanted components are both highly compromised in the presence of large metaphyseal and metaphyseo-diaphyseal bone defects. A stable Bone-implant construct enables correct alignment of the components, maintenance of adequate height for the joint line and ligament balance. Thus, the stability of the construct tailors the clinical outcomes and determines its longevity [1].

There have been some traditional surgical options that have been advocated to treat these bone defects and they include- utilization of bulk allografts [2,3], allograft impaction grafting [4,5], allograft-prosthetic composites [6] and custom/hinged prostheses [7].

Failure rates of almost 20% at 5 years have been documented by techniques like structural bone grafting [8]. It is still not known which is the best treatment strategy for these common revision TKA scenarios. Ever since the use of tantalum cones was started in the USA after the FDA approval,initial reports were published and technical nuances to use them were advocated [9,10]. Several other publications have followed up to help us know better about the efficacy and clinical outcomes pertaining to this biomaterial use in the management of large metaphyseal bone defects and they infact seem promising, although have documented only short term follow-ups [11,12].

Recently, the results of tantalum cones have been published by the Mayo group [13] with an intermediate follow-up study of about 5–9 years. For extensive metaphyseal defects, the use of Tantalum cones is usually combined with adjuvant stems which may be cemented/cementless and metallic bone augments for additional stability. These are metallic baseplate augments which are placed in a stacked fashion to build up the tibial joint surface to the nearest normal position. The tibial cones are press-fitted in the remnant metaphysis till they are flush with the large defect and the remaining height is reconstituted by augments.

However, there has been no published data till date that documents any scope of elimination of these extra metallic constructs by using a thicker tibial tantalum cone that builds up the tibial metaphysis directly to the base plate. There has not been a single study or case report sharing the rationale/validity of such a construct and its potential benefits/longevity.

We, thus, report a 2–6 year follow-up results of 6 patients, a Case series with large tibial metaphyseal defects(AORI type-3) in a revision TKA setting treated only with thicker tantalum cones forming single Baseplate-Cone construct and without augments in situations where augments were otherwise warranted. We would like to state that this Case series has been reported in line with the Process Statement [14].

## 2 Material and methods

The Tibial Baseplate-Cone Construct without Augments(BCCA) [We would like to advocate this terminology for such a construct where one can deliberately eliminate the use of augments by using a thicker cone and building up the joint surface to the nearest possible anatomical position] was used in 6 patients with AORI type-3 massive tibial metaphyseal bone loss encountered in Revision TKA. These cases were specifically those revisions that had defects where the augments would have been used otherwise to elevate the tibial joint surface to a closest native position. The surgeries were carried out by a single Senior surgeon (T.V.S.) at the same Institution between 2010-2016.

### 2.1 Patient demographics

Sex distribution was 4 males and 2 females. The average age at the time of surgery was 69 years with a range of 58–82 years. BMI was noted for all patients. Comorbid conditions amongst these patients were 2 patients with obesity, 1 had diabetes and 1 patient was a chronic smoker at the time of surgery.

### 2.2 Type of defect, previous surgeries and cause for revision

According to the AORI classification, all the defects were AORI Type-3 intraoperatively as the previous metal was explanted. Our patients had a prior average of at least 2 knee arthroplasty procedures elsewhere with all having a previous Stemmed tibia Revision prosthesis. The cause of Revision was aseptic in about 5 patients and 1 was a reimplantation as a part of the 2 stage Infected Revision protocol. The Patient demographics and other details have been tabulated in Table 1.

**Table 1:** XXX.

Patient No.	Age/Sex	BMI	Comorbid Conditions	Cause of Revision	AORI Defect Type 2B/3	Pre-op KSS score	Pre-op ROM (Flexion)	Post-op current KSS	ROM current
1.	68y/M	34.56	Obesity	Aseptic loosening	Type 3	45	80	80	100
2.	82y/F	25.05	None	Aseptic loosening	Type 3	52	70	86	110
3.	58y/M	28.7	None	Aseptic loosening	Type 3	56	90	78	110
4.	82/F	24.8	None	Aseptic loosening	Type 3	44	75	74	90
5.	65y/M	25.82	Smoking	Aseptic loosening	Type 3	57	80	87	115
6.	63y/M	37.60	Diabetes, Obesity	Septic loosening	Type 3	40	55	71	100

ROM = Range of motion.

KSS = Knee Society Score.

### 2.3 Surgical technique

The surgical technique was the usual technique used for the Tantalum cone construct with certain related differences. After removal of the previous prosthesis with most minimal iatrogenic bone loss, the bone defect was re-assessed intraoperatively and re-classified if evident as per the Anderson Orthopedic Research Institute Classification [5].

The host bone was prepared for the reception of the Cone with a high-speed burr. We tried to use the Cone that would press-fit to obtain maximum axial stability but at the same time reconstitute the joint line to the closest native position. After using trials and predicting a satisfactory reconstitution, the final cone was press-fitted and morsellized allograft bone/demineralized bone matrix (DBX; Synthes Westchester Pennsylvania) used to fill in any minute voids between the host bone and the cone so as to make efforts for maximal host bone-cone contact area. The femoral and tibial trials with the stems were used to determine and reconfirm native reconstitution of joint line and patellar tracking. The definitive components were then cemented to the tantalum cone. We cemented all our tibial stems upto the diaphysis (except one case where the cement extended only upto the metaphyseal area) and the tibial base-plate to the proximal surface of the cone. There was no contact between the baseplate and the host bone in all our cases and we took precautions to eliminate any chances of cement extravasating between the cone and the host-bone. Simplex P bone cement (Stryker, Mahwah, NJ) was used in all cases. As per our antibitotic protocol, we added 1gm of vancomycin and 1.2gm of tobramycin per batch of cement for all our revision cases.

### 2.4 Intraoperative peculiarities and construct characteristics

Intraoperatively, all patients required an adjuvant release procedure for better exposure of the knee (Quadriceps snip in 3 patients, Lateral releases in 2 others and just extended medial femoral tibial peel was enough in 1 patient).

The characteristics of the Construct are depicted in Table 2 with the

**Table 2:** XXX.

Patient No.	Prior Knee surgical procedures	Implant (Primary)	Implant (Revision)	Insert size	Stem length	Stem Cement (D/M)	Cone Type (S/U)	Cone height (mm)	Level of Constraint	Complications if any	Xray findings (present)	Total Follow up years	Revision free survival period	Results interpreted as E/G/F/P
**1.**	Two	Journey II	Smith n Nephew Legion Revision Tibia	12	120	D	U	30 mm	PS	None	SOINA	5	5(Operated for Patellar component aseptic loosening in 2014)	E
**2.**	Two	TC3 J n J	TC3 Mobile bearing	10	120	M	U	30 mm	PS	None	SOINA	2.5	2.5	E
**3.**	Multiple	Smith n Nephew Genesis-1 Revision component	Legion (Smith n Nephew) Revision Tibia	18	160	D	U	40mm (15 mm in contact with bone)	PS	None	SOINA	1.5	1.5	G
**4.**	Multiple	Genesis-2	Legion Tibia	14	160	D	U	15 mm	PS	None	SOINA	5	5	G
**5.**	Two	Genesis-2	Legion Tibia	10	120	D	U	30 mm	PS	Wound gaping(treated with secondary suturing)	SOINA	6	6	E
**6.**	Multiple	Genesis-2	Legion Tibia	10	120	D	U	30 mm (15 mm in contact with the bone)	PS	None	SOINA	5	5	G

D = Diaphyseal; M = Metaphyseal.

U = Unstepped; S = Stepped.

SOINA = Successful Osteointegration with no abnormal findings.

E/G/F/P = Excellent/Good/Fair/Poor.

1. Cement extent = diaphyseal/metaphyseal.

2. Type of cones = Stepped/Unstepped (all our cones were Unsteppped).

3. Type of implant (prior/revision) and Level of constraint (PS/Hinge) used.

4. Polyethylene insert size.

5. Stem length.

6. Tibial cone height.

Clinical follow up was done for each patient. The patients were assessed preoperatively to note the ROM and the Preoperative KSS. Postoperative ROM and Postoperative KSS have also been tabulated (Table 1).We did the radiological screening of X-rays on follow-up for signs of loosening, osteolysis and other abnormalities. Knee Society radiographic evaluation criteria modified for long-stemmed prostheses [15] were used to assess the integrity of the tibial stem-cone construct. Specific examination was performed of the cone-host bone interfaces for evidence of osteointegration. Complications if any (infection, fracture, resurgery) were documented. Revision free survival period for each patient was noted. Results were interpreted as excellent, good, fair, poor. Minimum clinico-radiological follow-up was 1.5 years (maximum being 6 years in one patient and 5 years in 2 patients).These have also been tabulated in Table 2.

## 3 Results

The results of these patients are tabulated in Table 1, Table 2.

100% osteointegration noted in final radiographs of all patients at an average follow-up of 4.1 years. Preoperative average ROM/KSS of 75/49 improved to postoperative ROM/KSS of 104/79.Outcomes were interpreted as excellent in 50% of cases and good in the remaining 50%.

**Fig. 1:**
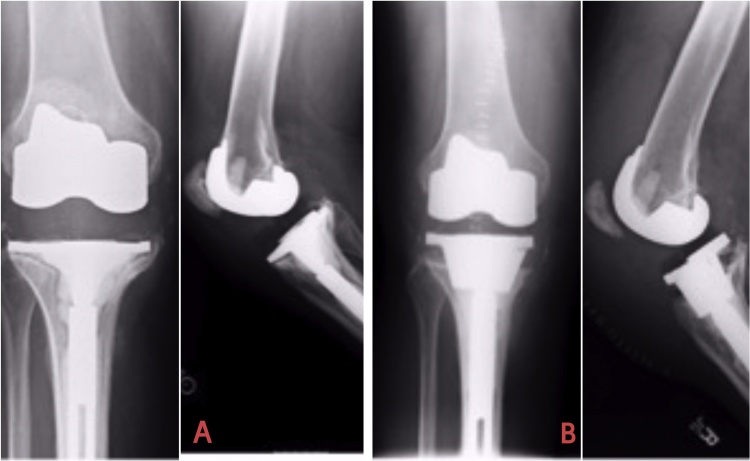
Fig. 1A=Preoperative Xrays of Journey II twice previously operated knee with aseptic osteolysis and loosening of the Revision Tibia component which was revised to Legion revision tibia with proud Tibial cone without augments. Fig. 1B depicts radiographs at 5 years follow-up.

**Fig. 2:**
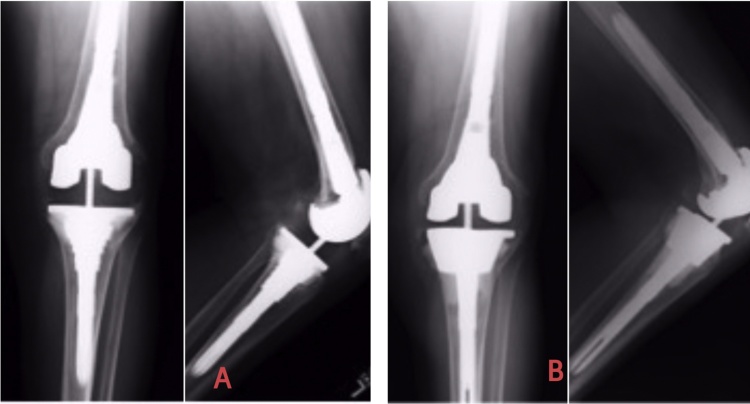
Fig. 2A shows the TC3 Johnson and Johnson Revision prosthesis with Tibial aseptic loosening (AORI Type-3) revised to the TC3 mobile bearing prosthesis with a 30 mm Tantalum cone (Fig. 2B).

**Fig. 3:**
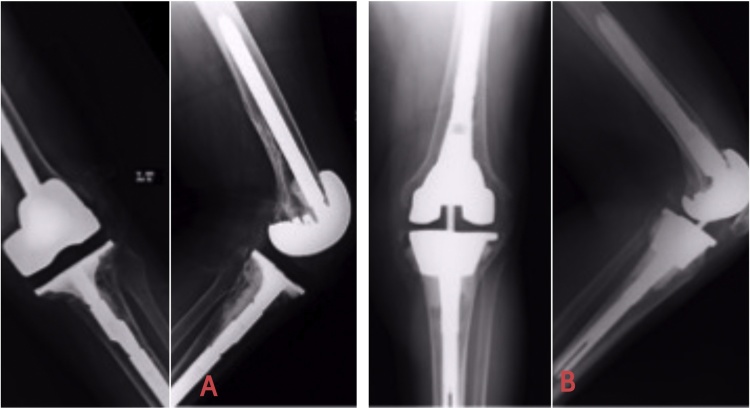
Fig. 3A show the Smith n Nephew Genesis Revision stemmed prosthesis with Type-3 defect which was later revised to Smith n Nephew Legion Revision stem with the unstepped Tantalum cone (Fig. 3B). Note that only 15 mm of the cone was in contact with the bone.

**Fig. 4:**
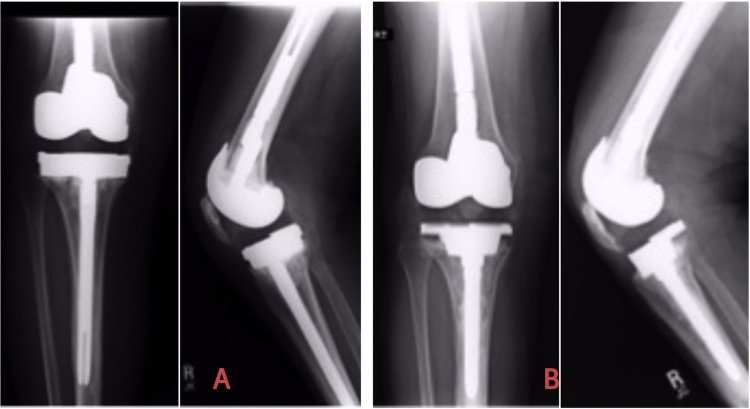
Fig. 4A illustrates the Genesis-2 augmented tibial tray which was revised for aseptic loosening. The defect got converted into an AORI type-3 after the explant of the Tibial tray. It was converted to the Legion Tibial tray with unstopped Tantalum metaphyseal cone (Fig. 4B).

**Fig. 5:**
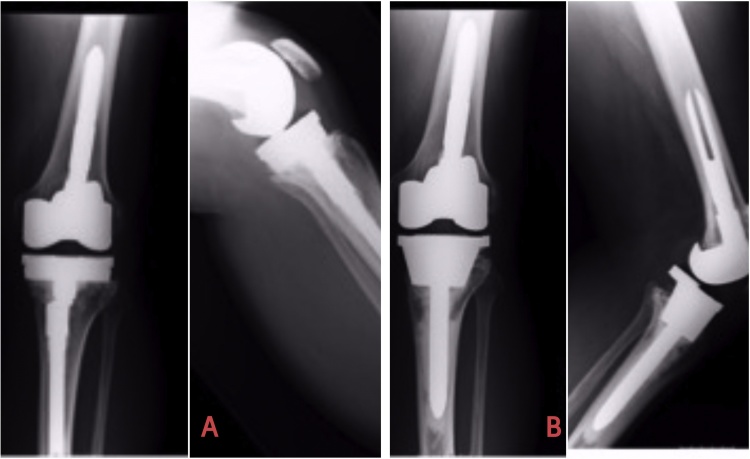
Fig. 5A shows the Genesis-2 tibial component with aseptic osteolyis and persistent pain. Note the thick metallic augment which leads to metaphyseal bone loss when explanted. This was revised to Legion tibial tray with only Tantalum cone without augment. Note the proud cone in the lateral view (Fig. 5B) and thus managing only 10 mm of Insert size (6 years F/U radiograph).

**Fig. 6:**
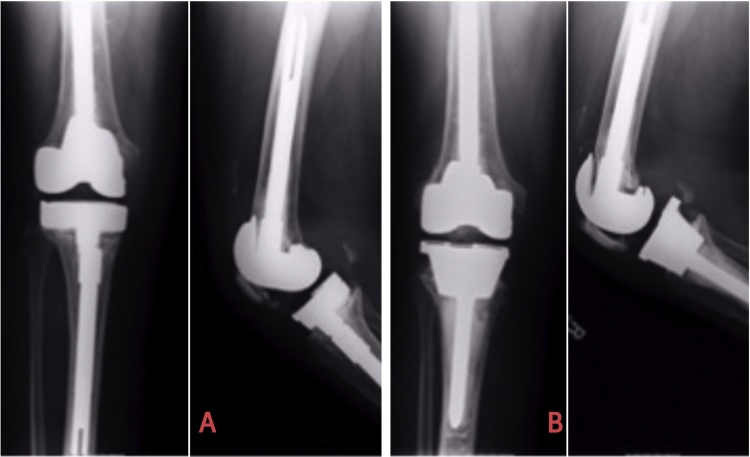
Fig. 6A illustrates the Genesis-2 tray with thick metallic augment subjected to multiple revisions previously (with septic loosening) and revised to a Legion PS tibial component with a 30 mm unstepped Tibial cone. Note that there was only 15 mm contact of Cone-Bone interface with a 5 year F/U Xray (Fig. 6B).

Radiographs of each patient are compiled in the figures with pre/post-op images. Figure number indicates each patient (numerically) with suffix A and B depicting preoperative (when presented to us) and postoperative radiographs (at final F/U) respectively. Years of F/U are stated in Table 2 for each patient with maximum of 6 years and minimum of 1.5 years (Fig. 1, Fig. 2, Fig. 3, Fig. 4, Fig. 5, Fig. 6).

## 4 Discussion & review of literature

There has gradually been an exponential increase in the overall burden of Revision Total knee replacements, many of which are complex with moderate to large tibial bone defects. The type of population that suffers from these complex bone defects is now a more active section of the society [16] with bone deficiencies that are a result of aseptic osteolysis secondary to particulate wear debris [17].Many of these lesions are extremely large and sometimes relatively asymptomatic [17,18] and it is difficult to gauge their extent till they are examined intraoperatively. Further bone loss may occur at the time of implant removal. Most times, they are underestimated [17,18] and can complicate preoperative planning, with less arrangements of predictive intraoperative armamentarium than is actually required.

The best treatment of such lesions has been a matter of debate since the inception of the concept of Total knee arthroplasty and the resultant options for managing this complication [9]. AORI Type 2B and Type 3 defects are one of the most problematic bone defects to treat during a revision setting. There have been various options that have been available to effectively manage these massive bone deficiencies like use of structural allograft [19], the combined use of impaction bone-grafting with mesh or the use of custom-designed implants. However, there have been advantages and disadvantages of each and the results have been far from satisfying. Also, metallic augments have not sufficed in treating these major defects. Even if they are used in majority of the complex knee revisions, they usually have to be supplemented by structural bulk allografts [19].

The studies available that have evaluated the safety and effectiveness of using homologous structural grafts from musculoskeletal tissue banks during revision surgery present limited numbers of cases and lack long-term postoperative follow-up [5,7], although short-term and medium term outcomes have been reported in small patient population [20]. Their proposed advantages have been their biologic in growth potential, versatility, relative cost-effectiveness, potential for bone stock restoration, and potential for ligamentous reattachment and their ability to unite to the host bone with a poor cancellous structure [21].

The disadvantages of structural allografts include the risk of disease transmission, nonunion/malunion, collapse or resorption of the graft and the meticulous preparation required to maximize surface contact between the allograft and the host-bone interfaces [21]. Repeat revisions with these construct types have been reported in 23% in one multicenter study [22] and 9% in another report [3]. Backenstein et al. [23] reported a failure rate of 21.3% and Bauman RD et al. [8] proposed a 22.8% revision rate.

A recent valid addition to these varied treatment options has been the use of Tantalum metal cones and its proposition as the closest biocompatible material to treat these extensive bone defects [10]. There have been several properties of this metal like the high coefficient of friction, low elastic modulus and high porosity that make it an ideal bone substitute [24]. There are also proposed helpful scaffolding properties for osteoblastic activity [25]. These characteristics help gain excellent initial mechanical stability, enables better load distribution(akin spongy bone), reduces stress-shielding, enables biological osteointegration and cement interdigitation. Clinical advantages that translate from these properties include successful bone ingrowth despite deficient host bone [26], predictive better remodeling potential similar to tantalum cups in hip arthroplasty [27],lack of late collapse seen with allografts, no risk of disease transmission, lower infection risk, simplified surgical technique as less host bone preparation and immediate weight bearing postoperatively [26].

Matching of these modular cones to the varied defects of different sizes and shapes is easier than matching a bulk allograft to the host bone. The only proposed disadvantage of tantalum cones is its anticipated difficult removal, if need be for an infection revision because of its potential exuberant in growth potential [9]. Several small clinical studies have demonstrated encouraging results with Tantalum cones [[9], [10], [11], [12], [13]]. A recent intermediate follow-up study has in fact reinforced the initial enthusiasm that was kick-started by other previous short-term follow-up literature [13].

In the last 10 years of the author’s practice, there has been extensive use of tantalum cones in revision TKAs where reconstitution of AORI Type 2B/3 defects was warranted with excellent clinical outcomes in all patients. By reporting this case series of 6 patients treated with cones that were kept proud to eliminate the need for using adjuvant metallic augments, we have attempted to go one step ahead in the technical application of this excellent biomaterial. Our average follow-up of these patients demonstrate good results for using larger tantalum cones which stand out beyond the metaphyseal tibial sleeve of host bone and thus, exclude the addition of an extra mechanical construct interface between the tibial base plate and the metallic augments. Also, based on the generally accepted principle of using shorter cemented stems with tantalum cones instead of longer diaphyseal fixation stems for early implant fixation and antibiotic elution [13],we used shorter cemented stems in all the reported cases. Another proposed advantage in such a situation could be stronger Tantalum cone-cement interdigitation around the stem due to a longer contact area between them because of exclusion of metallic augments. Also, the tibial tray baseplate sitting directly on the cone with interfingering cement could be another parallel boost to the construct stability over the base plate-augment interface with smooth metallic surfaces on both sides. A valid drawback of this case series report is a small sample size and an intermediate follow-up (in 4 patients and short-term follow-up in 2 patients).

## 5 Conclusion

It will be worthwhile to know whether this “Tibial base plate-cone construct without Augments (BCCA)” type of a construct (Swanson’s BCCA technique) has any valid long term advantage over the Tibial base plate-Augment-Cone combination in massive tibial bone defects. Larger cohort based multi-center studies would be required to explore this concept and its proposed technical advantage. Also, it would be intriguing to know if these concepts can also be applied for similar situations on the femoral side. Although we are presenting a small series of patients, the results seem to be encouraging and reinforce the already enthusiastic outcomes of the use of Tantalum cones in the treatment of AORI Type 2A/3 massive metaphyseal bone defects.

## Conflicts of interest

None.

## Funding source

None.

## Ethical approval

Our Institute exempted our Case series surgical technique study from the need of any Ethical approval.

## Consent

Written informed consent was obtained from the patients for publication of this case report and accompanying images. A copy of the written consent is available for review by the Editor-in-Chief of this journal on request

## Author contribution

Dr. Mohit Kukreja- Data collection, Patient interviewing, KSS scoring, Manuscript writing and Literature collection.

Dr. Todd V. Swanson- Manuscript editing, Proof reading, Bibliography refining and final Agreement.

## Registration of research studies

UIN .4191 from Research registry.

## Guarantor

Dr. Mohit M. Kukreja.

## Provenance and peer review

Not commissioned, externally peer-reviewed.
